# Enhanced HIFU Theranostics with Dual-Frequency-Ring Focused Ultrasound and Activatable Perfluoropentane-Loaded Polymer Nanoparticles

**DOI:** 10.3390/mi12111324

**Published:** 2021-10-28

**Authors:** Junjie Chen, Zhezhu Nan, Yubo Zhao, Lei Zhang, Hongrui Zhu, Daocheng Wu, Yujin Zong, Mingzhu Lu, Tali Ilovitsh, Mingxi Wan, Kai Yan, Yi Feng

**Affiliations:** 1 The Key Laboratory of Biomedical Information Engineering of Ministry of Education, Department of Biomedical Engineering, School of Life Science and Technology, Xi′an Jiaotong University, Xi′an 710049, China; cjj2151200038@stu.xjtu.edu.cn (J.C.); nanzhezhu@stu.xjtu.edu.cn (Z.N.); zhugejiangwei@stu.xjtu.edu.cn (Y.Z.); zhlei012@xjtu.edu.cn (L.Z.); ilonce@stu.xjtu.edu.cn (H.Z.); wudaocheng@mail.xjtu.edu.cn (D.W.); yjzong@xjtu.edu.cn (Y.Z.); mzlu@mail.xjtu.edu.cn (M.L.); mxwan@mail.xjtu.edu.cn (M.W.); 2 Department of Biomedical Engineering, Tel Aviv University, Tel Aviv 6997801, Israel; ilovitsh@tauex.tau.ac.il; 3 College of Bioresources Chemical and Materials Engineering, Shaanxi University of Science and Technology, Xi’an 710021, China

**Keywords:** high-intensity focused ultrasound (HIFU), cavitation, theranostics, dual frequency, perfluoropentane-loaded polymer nanoparticle (PFP@Polymer NP)

## Abstract

High-intensity focused ultrasound (HIFU) has been widely used in tumor ablation in clinical settings. Meanwhile, there is great potential to increase the therapeutic efficiency of temporary cavitation due to enhanced thermal effects and combined mechanical effects from nonlinear vibration and collapse of the microbubbles. In this study, dual-frequency (1.1 and 5 MHz) HIFU was used to produce acoustic droplet vaporization (ADV) microbubbles from activatable perfluoropentane-loaded polymer nanoparticles (PFP@Polymer NPs), which increased the therapeutic outcome of the HIFU and helped realize tumor theranostics with ultrasound contrast imaging. Combined with PFP@Polymer NPs, dual-frequency HIFU changed the shape of the damage lesion and reduced the acoustic intensity threshold of thermal damage significantly, from 216.86 to 62.38 W/cm^2^. It produced a nearly 20 °C temperature increase in half the irradiation time and exhibited a higher tumor inhibition rate (84.5% ± 3.4%) at a low acoustic intensity (1.1 MHz: 23.77 W/cm^2^; 5 MHz: 0.35 W/cm^2^) in vitro than the single-frequency HIFU (60.2% ± 11.9%). Moreover, compared with the traditional PFP@BSA NDs, PFP@Polymer NPs showed higher anti-tumor efficacy (81.13% vs. 69.34%; * *p* < 0.05) and better contrast-enhanced ultrasound (CEUS) imaging ability (gray value of 57.53 vs. 30.67; **** *p* < 0.0001), probably benefitting from its uniform and stable structure. It showed potential as a highly efficient tumor theranostics approach based on dual-frequency HIFU and activatable PFP@Polymer NPs.

## 1. Introduction

High-intensity focused ultrasound (HIFU), also known as focused ultrasound surgery (FUS), is a noninvasive method for the treatment of lesions located deep within the body. In HIFU treatment, the ultrasound beam propagates through soft tissue as a high-intensity pressure wave focused on a small target volume, converting energy into heat at this location. An increase in the temperature of the target tissue causes coagulative necrosis and protein denaturation within seconds. By moving the HIFU focus region, larger target tissues can be ablated with millimeter accuracy [1]. HIFU can provide completely noninvasive treatment without causing damage to the adjacent tissues [2]. As a result of its deep penetration of soft tissue and good therapeutic efficiency, HIFU ablation has been a promising alternative to invasive surgery for the treatment of several malignant or benign diseases located in the superficial or deep layers of the body, e.g., uterine fibroids, tumors of the prostate, breast, liver, and brain (transcranial) [1,3,4,5]. However, real-time imaging-guided detection by HIFU is still difficult to achieve, especially with the thermal effects to be detected [6]. To date, several medical imaging protocols, e.g., X-ray/CT, ultrasound (US), and MRI [7,8,9], have been used. Among them, the contrast-enhanced ultrasound (CEUS) imaging has a great advantage in monitoring cavitation events over short timescales [10,11]. When HIFU ablation works on the phase transition of nanodroplets into microbubbles, CEUS imaging shows a great potential in monitoring the HIFU-activated microbubbles in real time [12,13,14].

The current results of HIFU treatment are still unsatisfactory. For instance, it is difficult for tumor tissue rich in blood supply to reach the treatment temperature without side effects due to the acoustic window limits and over-vascularization, and the residual tumor cells after thermal ablation may result in tumor recurrence [15]. Moreover, HIFU treatment may cause serious complications, such as skin burns and liver and abdominal abscesses due to its high energy output [16,17,18]. Cavitation can enhance the efficacy of HIFU through its thermal [19,20], mechanical [21], and chemical effects [22,23]. Therefore, the synergistic effect of cavitation and HIFU is considered to be a promising way to enhance the therapeutic outcomes. Improving the cavitation effect under imaging guidance is a feasible way to reduce the energy output of HIFU and improve its efficacy.

The introduction of cavitation nuclei, such as exogenous ultrasound contrast agents, is an effective way to reduce the cavitation threshold and improve the cavitation effect. Among them, nanodroplets (NDs) present some advantages over microbubbles (MBs) for tumor treatment, such as a longer in vivo lifetime by avoiding rapid renal clearance and reticuloendothelial system uptake, higher vascular extravasation by the enhanced permeability and retention (EPR) effect, and higher intracellular uptake by endocytosis [24]. Some fluorocarbon micro- and nanodroplets locally enhance the thermal and cavitation effects of HIFU therapy, achieving more accurate, effective, and safe soft tissue ablation [25]. Two mechanisms are mainly involved. One is acoustic droplet vaporization (ADV), in which focused ultrasound above the threshold of rarefied pressure is used to convert the perfluorocarbon droplets into microbubbles [26,27,28]. In the other mechanism, the microbubbles formed by the vaporization of NDs can further perform the inertial cavitation (IC) process under ultrasound irradiation and achieve enhanced ultrasonic monitoring imaging [29]. Choi et al. [30] cross-linked six-arm-branched poly(ethylene glycol) (PEG) with Pluronic F127 mixed with naphthalocyanine (Nc) and sonicated it with liquid perfluorohexane (PFH), which formed nanoparticles encapsulating Nc and PFH (Nc/PFH@PCPN), enhancing the therapeutic efficacy of high-intensity focused ultrasound (HIFU) treatment and image guidance by photoacoustic (PA) and ultrasound (US) imaging. Lee et al. [31] prepared PEGylated mesoporous silicatitania nanoparticles (P-MSTNs) used as US-responsive nanocarriers for cancer sonotheranostics. Perfluorohexane (PFH), chosen as the gas precursor, was physically encapsulated in P-MSTNs using the oil-in-water emulsion method. These nanodroplets showed both an enhanced cavitation effect and excellent ultrasonic imaging performance.

Moreover, we have proved that dual-frequency ultrasound can reduce the threshold and improve the efficiency of ADV and IC [32]. Dual-frequency excitation is an easy and effective approach to increasing the size of HIFU-produced lesions and, subsequently, decreasing the treatment time of cancer and solid tumors [33]. Enhanced bubble dynamics and the associated heating enhancement play a major role in this enhancement mechanism. More cavitation activities were also confirmed at the dual-frequency excitation, which may improve the performance of HIFU ablation [33]. In addition, studies have shown that tissue ablation using dual-frequency HIFU yield a higher temperature and a higher temperature rise rate compared with ablation using single-frequency HIFU under the same exposure power and time [34].

**Scheme 1:**
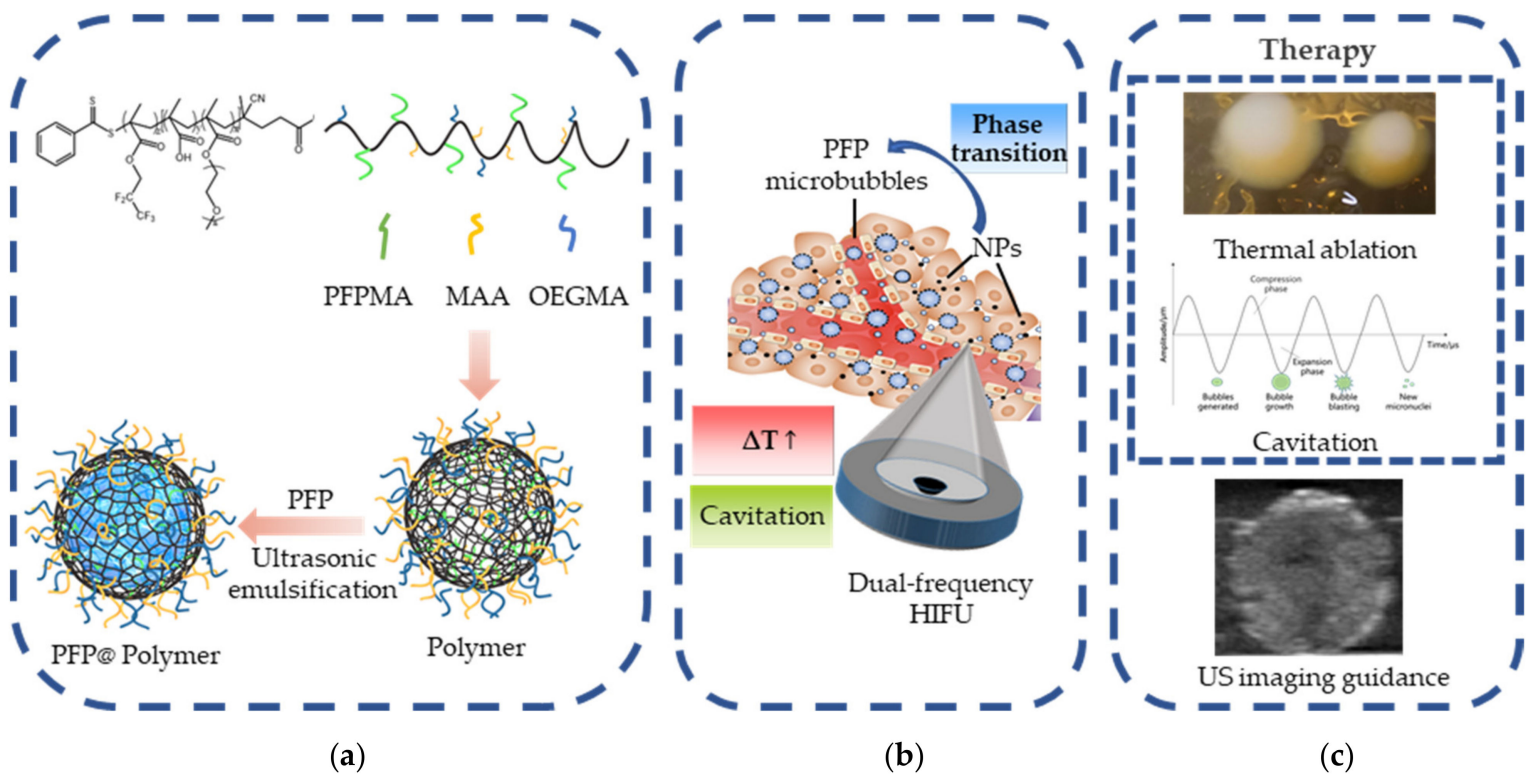
Schematic illustration showing (**a**) the synthetic process of theranostic PFP@Polymer NDs, (**b**) mechanisms of thermal and cavitation-enhanced cancer therapy via the PFP@Polymer NDs irradiated by dual-frequency HIFU, and (**c**) thermal ablation and cavitation effects guided by US imaging.

In our previous study, we designed and prepared a one-for-all nanodroplet encapsulated by polypyrrole (PFP@Ppy ND) and achieved highly efficient US-imaging-guided and cavitation-enhanced photothermal therapy. Remarkable diagnostic and therapeutic effects have been obtained in the combined photothermal–cavitation therapy in vivo and in vitro [29]. However, photothermal therapy has some limitations, such as low penetration depth and inability to treat deep tumors. Energy management also needs to integrate two instrumental systems, laser and US confocal, and requires synchronization control, making the theranostics system too complicated. Therefore, in the present study, HIFU thermal ablation was used instead of near-infrared laser to overcome the limitation of its penetration depth and simplify the instrumental system. We designed and prepared fluorinated-polymeric-nanomicelle-loaded perfluoropentane (PFP) nanoparticles (PFP@Polymer NPs), as shown in Scheme 1. Dual-frequency (1.1 and 5 MHz) HIFU was also introduced to promote the formation of ADV microbubbles and cavitation-enhanced HIFU therapy. With the help of PFP@Polymer NPs, the thermal effect from the lower (1.1 MHz) component of dual-frequency HIFU can directly cause coagulative necrosis of tumor cells and activate the phase transition of PFP into microbubbles with the assistance of cavitation activation effect from the higher (5 MHz) component of dual-frequency HIFU. The liquid–gas boundary of microbubbles, as the strong reflection interface of ultrasound, can further enhance the energy absorption of tissue and improve the efficiency of heat production. The mechanical effect, the transient high temperature, high pressure, and ROS caused by the IC of microbubbles can improve the therapeutic outcome of HIFU. Furthermore, HIFU-activated microbubbles have the potential to work as contrast agents for CEUS imaging to guide the treatment.

## 2. Materials and Methods

### 2.1. Materials

4-Cyano-4-(thiobenzoylthio)pentanoic acid (CTPBA); 1H, 1H-pentafluoro-N-propyl methacrylate (PFPMA); methacrylic acid (MAA); poly(ethylene glycol) methyl ether methacrylate (OEGMA); azodiisobutyronitrile (AIBN); perfluoropentane (PFP); and bovine serum albumin (BSA) were purchased from Sigma-Aldrich (USA); CCK-8 and RPMI 1640 medium were purchased from Mishushengwu Biotechnology Co., Ltd. (Xi’an, China); FBS was purchased from Sijiqing Biological Engineering Materials Co., Ltd. (Hangzhou, China); and tetrahydrofuran (THF) was purchased from Meryer Chemical Technology Co., Ltd. (Shanghai, China). All reagents were used as received without additional treatment.

### 2.2. Ultrasound Apparatus

**Figure 1:**
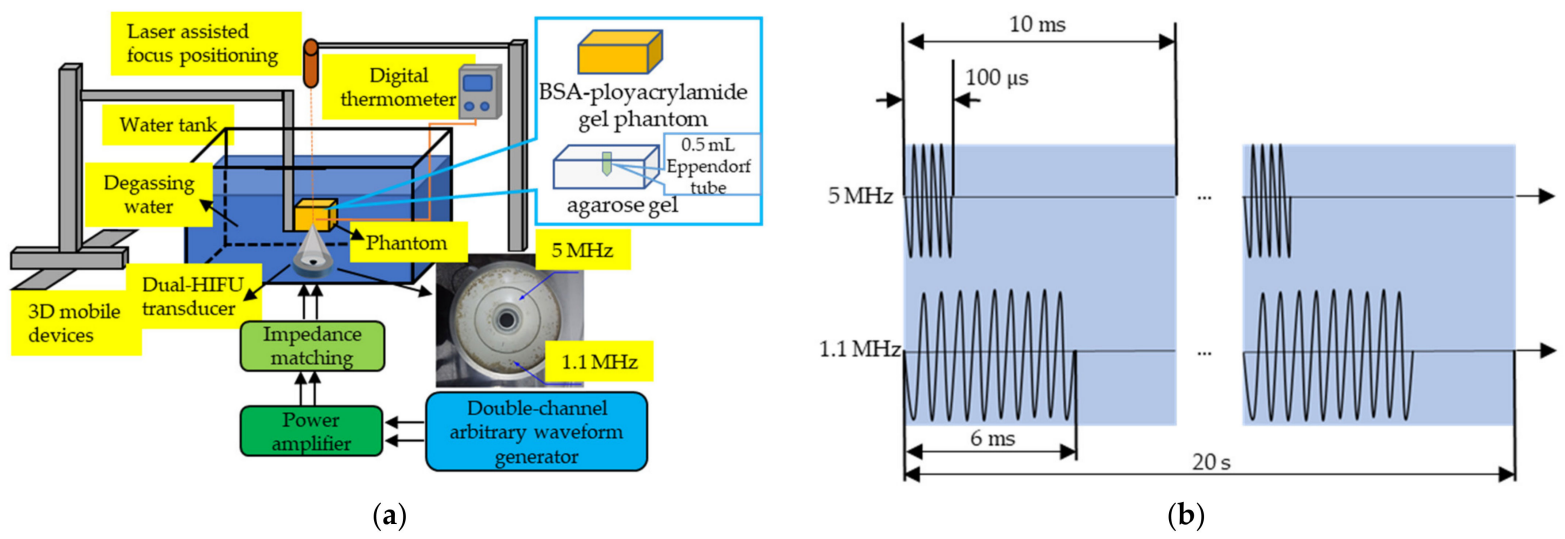
Experimental setup (**a**) and excitation waveform (**b**).

Transmitted pulses were generated by a custom-designed transducer (Chongqing Haifu Medical Tech. Co., Ltd., Chongqing, China) consisting of two confocal transducers. Both transducers were spherical annular, one was stimulated at 1.1 MHz (outer) and the other at 5 MHz (inner). The diameters of the outer (1.1 MHz) ring transducer and the inner 5 MHz ring transducer were 94 mm and 50 mm, respectively. Two ring transducers were confocal at a focal distance of 60 mm. The experimental setup is shown in Figure 1a. The driving signals for elements of the annular transducer were generated by a double-channel arbitrary waveform generator (DG5072, RIGOL, Beijing, China) and amplified by two power amplifiers (AG1016, T&G Power Conversion, Inc, Rochester, NY, USA). The transmission pulse duration was 100 μs. The duty cycle of the low-frequency (1.1 MHz) transducers was 60% and that of high-frequency (5 MHz) transducers was 1%, which mainly contributed to the thermal effect and the cavitation effect, respectively. The two transducers were triggered with the same pulse repetition frequency of 100 Hz. For the in vitro experiments, 4T1 cells were placed in the confocal zone of the dual-frequency ultrasound transducer surrounded by the degassed water to facilitate ultrasound transmission. The temperature was monitored by a digital thermometer (Shenzhen Anseny Electronic Technology Co., Ltd.) before, during, and after US treatment. The needle thermocouple was inserted in the phantom horizontally, with its top placed at 2 mm right to the HIFU confocal point. The acoustic intensity corresponding to the electric power for 1.1 and 5 MHz HIFU in the experiment are shown in Table 1 and Table 2.

### 2.3. Synthesis and Characterization of Activatable PFP@Polymer Nanoparticles

OEGMA (1.4 g), MAA (0.86 g), AIBN (3.28 mg), and CTBPA (28 mg) were dissolved in THF in a 30 mL flask equipped with a magnetic stirring bar. The mixture was subjected to 68 °C for 5 h for RAFT polymerization. After that, the mixture was precipitated in hexane and redissolved in THF. The purification steps were carried out three times. The poly(OEGMA-co-MAA) copolymer was obtained in a vacuum oven overnight at 40 °C. Then, poly(OEGMA-co-MAA) (1 g), PFPMA (0.8 g), and AIBN (3 mg) were charged into a 30 mL flask. The flask was degassed by three freeze–pump–thaw cycles and sealed under vacuum. After polymerizing for 24 h at 70 °C, the mixture was also precipitated into hexane and redissolved in THF. Finally, poly(OEGMA-co-MAA)-b-PFPMA block copolymer was acquired as a powder.

The polymer nanomicelles were prepared as follows: 50 mg of poly(OEGMA-co-MAA)-b-PFPMA copolymer was add to 0.5 mL of THF, which was followed by the addition of 15 mL of degassing water. The mixture was cooled down in an ice bath. Then, 300 μL (2% *v*/*v*) of PFP liquid was mixed in, and the mixture was ultrasound-sonicated in an ice bath for 2 min to get a PFP@Polymer nanoparticles emulsion, which could be stored at 4 °C. The structural morphology of the PFP@Polymer nanoparticles was evaluated by transmission electron microscopy (TEM). The size distribution was determined by dynamic light scattering (DLS).

### 2.4. Cell Culture

The murine breast cancer cell line 4T1 was maintained in an RPMI 1640 medium supplemented with 15% FBS, at 37 °C in a humidified incubator with 5% CO_2_. Cell viability was >98% as assessed with the trypan blue exclusion test before each treatment.

### 2.5. In Vitro Cytotoxicity Evaluation

To evaluate the cytotoxicity of the PFP@Polymer NPs, 4T1 cells were incubated in 96-well plates with the PFP@Polymer NPs at volume concentrations of 0.0002–0.02% (*v*/*v*) for 24 h, and then, a cell counting kit assay (CCK-8, Mishushengwu, Xi’an, China) was used to evaluate the cell viability according to the manufacturer’s instructions. Briefly, a 10% (*v*/*v*) CCK-8 solution was added to the culture medium and incubated for 1 h. The absorbance at 450 nm was measured using a microplate reader (SpectraMax 190, Molecular Devices, San Jose, CA, USA), and the cell viability was calculated as we described previously [29].

### 2.6. Evaluation of Thermal and Cavitation Effects in the Biomimetic Phantom

The transparent BSA–polyacrylamide gel phantom has acoustic characteristics similar to those of soft tissue and a good ability to indicate any thermal injury [35]. In this study, we used the BSA–polyacrylamide gel phantoms with and without NPs to verify the thermal and cavitation effects of dual-frequency HIFU and activatable PFP@Polymer NPs on treatment. The BSA–polyacrylamide gel phantoms were prepared as we previously described [36]. To prepare the biomimetic phantom containing NPs, 0.002% NPs was added. The heat denaturation of BSA produces milk-white lesions in the phantom, which indicates the thermal lesions of HIFU. Cavitation of PFP@Polymer NPs could also be observed as a microbubble cloud.

The thermal and cavitation effects of single- and dual-frequency HIFU with and without PFP@Polymer NPs were measured and compared. We used a series of power gradient single-frequency 1.1 MHz (10–140 W; intervals of 10 W), 5 MHz (10–60 W; intervals of 10 W), and dual-frequency US treatment with different irradiation times (20 s, 30 s, 40 s, and 50 s) to evaluate the enhanced thermal and cavitation effects of dual-frequency HIFU and the activatable PFP@Polymer NPs and optimize the US parameters. The temperature in the phantom was also recorded during the ultrasound sonication to further verify the thermal effects of dual-frequency HIFU and activatable PFP@Polymer NPs.

### 2.7. Evaluation of Anti-Tumor Efficiency of Dual-Frequency HIFU and Activatable PFP@Polymer NPs

The anti-tumor effect of PFP@Polymer NPs activated by dual-frequency HIFU irradiation in vitro was evaluated. First, 4T1 cells ($2\times 10^{6}$ cells/mL) in the exponential phase were collected and incubated in a serum-free RPMI-1640 medium and then divided randomly into 10 groups: (1) control, (2) only NPs (0.002% *v*/*v*), (3) 1.1 + 5 MHz US (10 + 10 W), (4) NPs + 1.1 MHz US (10 W), (5) NPs + 5 MHz US (10 W), (6) NPs + 1.1 MHz US + 5 MHz US (10 + 10 W), (7) 1.1 + 5 MHz US (20 + 10 W), (8) NPs + 1.1 MHz US + 5 MHz US (20 + 10 W), (9) 1.1 + 5 MHz US (30 + 10 W), and (10) NPs + 1.1 MHz US + 5 MHz US (30 + 10 W). Each group was divided into three parallel groups, suspended in a sterile 0.5 mL Eppendorf tube, sealed with parafilm, and placed in a prepared 1% agarose gel holder for ultrasonic irradiation, as the previous study described [37]. After ultrasonic irradiation, the solution of each tube was transferred to a 96-well plate, 100 μL per well, for the following CCK-8 test. Here, 5 MHz US was fixed at the power of 10 W because it caused no significant damage lesions in the phantom even at the high power of 60 W. The power of 1.1 MHz HIFU in dual-frequency HIFU was set at 10, 20, and 30 W to compare the cell viability of single- and dual-frequency HIFU irradiation with and without NPs for 20 s. We also compared the in vitro single-frequency and dual-frequency therapeutic effects of common PFP@BSA NDs and PFP@Polymer NPs (0.002% *v*/*v*). The three groups were (1) the blank without adding any NDs or NPs, (2) the group with added PFP@BSA, and (3) the group with added PFP@Polymer NPs. Each group faced four different levels of ultrasound irradiation: (1) no US, (2) only 1.1 MHz US (10 W), (3) only 5 MHz US (10 W), and (4) 1.1 + 5 MHz US (10 + 10 W). The ultrasound irradiation time was 20 s.

### 2.8. Evaluation of the Enhanced Ultrasound Imaging In Vitro

Finally, we evaluated the ultrasonic imaging ability of the prepared PFP@Polymer NPs and the widely used PFP nanodroplets with a BSA shell (PFP@BSA NDs) after they were activated by 1.1 MHz HIFU irradiation, 5 MHz HIFU irradiation, and 1.1 + 5 MHz dual-frequency HIFU irradiation. Two kinds of nanosystems with the same concentration (0.002% *v*/*v*) of 2 mL were placed in a six-pore 1% agarose gel phantom (pore diameter 1 cm and depth 3 cm). Single frequencies of 1.1 and 5 MHz and dual-frequency interaction (10 W; irradiation time of 20 s) were used to irradiate PFP@Polymer NPs and PFP@BSA NDs, respectively. Then, all samples were imaged with the same settings using a diagnostic US scanner (SonixTouch, Ultrasonix, Richmond, Canada) in conventional B-mode and contrast-enhanced ultrasound (CEUS) mode (linear probe, 10 MHz, L40-8/12). The mean US intensity in the region of interest was determined by Image J software (NIH, http://rsb.info.nih.gov/ij/, accessed on 25 October 2020).

### 2.9. Statistical Analysis

Data were presented as the mean ± SD, and the statistical significance of the treatment outcomes were assessed using one-way ANOVA analysis of variance (GraphPad Prism software, version 8.0). * *p* < 0.05, ** *p* < 0.01, *** *p* < 0.001, and **** *p* < 0.0001 were considered statistically significant in figures.

## 3. Results

### 3.1. Characterization of Activatable PFP@Polymer Nanoparticles

**Figure 2:**
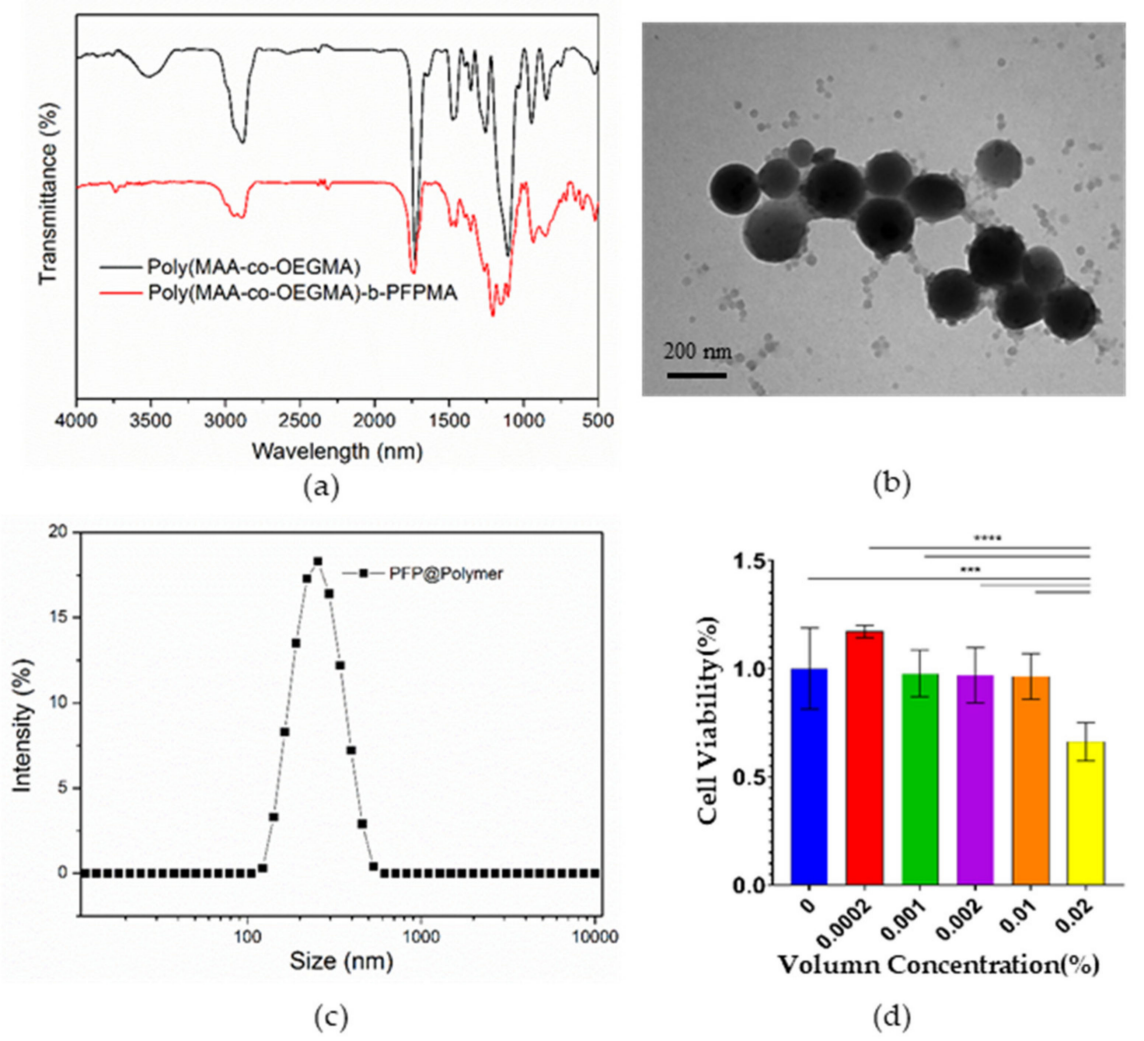
Characteristics of PFP@Polymer NPs. (**a**) FT-IR spectra of a polymer. (**b**) TEM image. (**c**) The size distribution. (**d**) Cytotoxicity of PFP@Polymer nanoparticles at different volume concentrations.

The FT-IR spectra of poly(MAA-co-OEGMA) and poly(MAA-co-OEGMA)-b-PFPMA are shown in Figure 2a. In the poly(MAA-co-OEGMA) spectrum, the characteristic peak located at 1737 cm^−1^ is associated with ester carbonyl (C=O) bands in MAA and OEGMA. The strong peak at 1104 cm^−1^ belongs to the ether bands (C-O-C) in OEGMA. This absorption of MAA and OEGMA can also be observed in the poly(MAA-co-OEGMA)-b-PFPMA spectrum. In addition, the characteristic peaks located at 1150 cm^−1^ are ascribed to the C-F groups. The strong peak at 2880 cm^−1^ arises from −CH_3_ and −CH_2_ groups. The fluorinated segments of polymers have good compatibility with perfluoropentane, so hydrophobic perfluoropentane was encapsulated by fluorinated polymer while the hydrophilic OEGMA and MAA segments stretched toward the aqueous phase, as shown in Figure 2b. The size of PFP@Polymer NPs is about 200 nm, which is in accordance with the DLS result (Figure 2c). Furthermore, PFP@Polymer NPs showed no significant cytotoxicity at the volume concentrations (*v*/*v*) of 0.0002% to 0.01%, proving their good biocompatibility (Figure 2d). 

### 3.2. Evaluation of Thermal and Cavitation Effects in the Biomimetic Phantom

**Figure 3:**
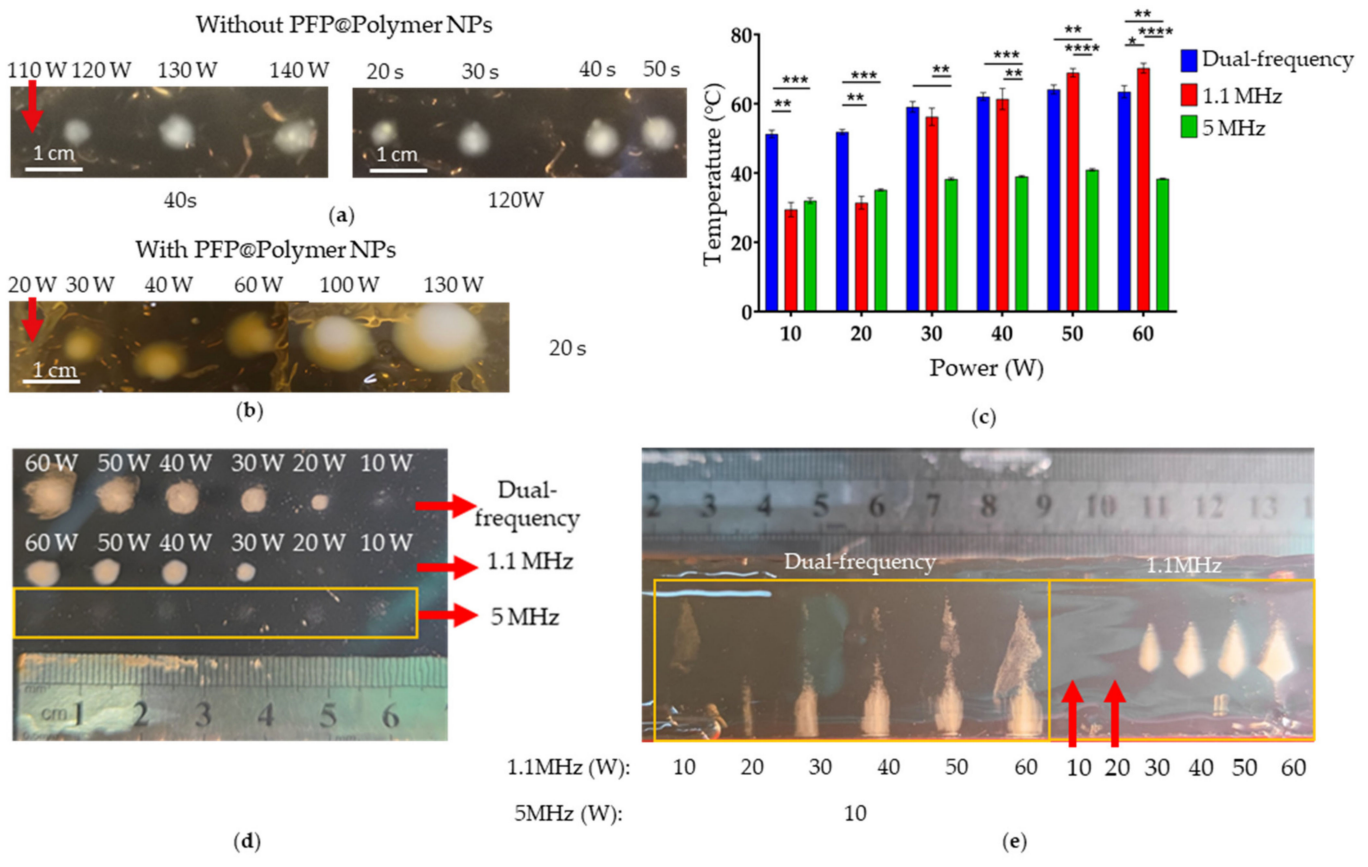
The enhanced performance of PFP@Polymer NPs and dual-frequency HIFU in the treatment in vitro. The damage effect without (**a**) and with (**b**) PFP@Polymer NPs in a BSA–polyacrylamide gel phantom under single-frequency 1.1 MHz HIFU irradiation (power of single-frequency 1.1 MHz HIFU: 20 140 W; intervals of 10 W. Irradiation time 20 s, 30 s, 40 s, and 50 s). (**c**) The temperature rise after single-frequency (1.1 or 5 MHz) and dual-frequency HIFU irradiation (power: 10 – 60 W; intervals of 10 W. Irradiation time: 20 s). When irradiated by dual-frequency HIFU, only the 1.1 MHz HIFU power is changed, and the 5 MHz HIFU power is fixed at 10 W. The water temperature is 25 °C. (**d**) The damage effect of PFP@Polymer NPs in BSA–polyacrylamide gel phantoms under 1.1 MHz, 5 MHz, and dual-frequency (1.1 + 5 MHz) HIFU irradiation and (**e**) its side view. * *p* < 0.05, ** *p* < 0.01, *** *p* < 0.001, and **** *p* < 0.0001.

To evaluate the effects of PFP@Polymer NPs on the energy threshold of HIFU damage, the damage of BSA–polyacrylamide gel phantoms with and without NPs on 1.1 MHz HIFU irradiation at different powers was recorded and compared in Table 3 and Figure 3a. When there were no NPs, irradiation of 1.1 MHz HIFU at 110 W for 40 s caused no damage lesions. However, the power was reduced to 30 W, and the time was shortened to 20 s to produce significant damage lesions with PFP@Polymer NPs. Moreover, compared with irradiation without NPs, the damage lesions increased by 175% in diameter on irradiation with NPs at the same power, as shown in Figure 3b. We also compared the power threshold of damage caused by single-frequency HIFU irradiation (1.1 or 5 MHz) and dual-frequency (1.1 and 5 MHz) combined irradiation, as shown in Table 4 and Figure 3d. There was no obvious damage even when the power of single-frequency 5 MHz HIFU was set to 60 W, and the obvious damage lesions could only be produced when the power of single-frequency 1.1 MHz HIFU was set to more than 30 W. However, dual-frequency HIFU (with the 5 MHz component fixed at 10 W) can produce a slight damage at 10 W of 1.1 MHz HIFU and obvious damage at 20 W. This proves that both dual-frequency HIFU and the PFP@Polymer NPs decrease the threshold of HIFU thermal ablation significantly.

The damage lesions caused by dual-frequency HIFU changed their shape and were 1.5–2 times larger than the lesions caused by single-frequency 1.1 MHz HIFU at the same power. Figure 3e shows a side view of damage lesions produced by single-frequency 1.1 MHz and dual-frequency HIFU, respectively. The thermal damage lesions (milk-white spot) produced by single-frequency 1.1 MHz irradiation are teardrop shaped. Under dual-frequency irradiation, the thermal damage lesions became larger, with a cylindrical shape, and moved toward the transducer. Above the damage lesions, a significant tapered thin cavitation bubble cloud was observed. Moreover, the higher the 1.1 MHz HIFU power, the larger the damage volume produced at the bottom, and the more obvious the cavitation bubble cloud in the top conical area. The possible reason for this phenomenon was that more cavitation bubbles in the focal region are produced by dual-frequency HIFU irradiation to form a strong reflection, which hinders the propagation of 1.1 MHz HIFU. Then, the reflected sound wave changes the energy distribution of 1.1 MHz, which makes the area with higher acoustic intensity of 1.1 MHz HIFU move toward the transducer, producing the thermal damage area with a different shape and at a certain distance closer to the transducer.

The temperature in the phantom was also recorded during the ultrasound irradiation to further verify the thermal effects of dual-frequency HIFU and activatable PFP@Polymer NPs, as shown as Figure 3c. At 30 W or more, single 1.1 MHz HIFU increased the temperature significantly, while 5 MHz HIFU showed no obvious effects on temperature rise even at 60 W. However, dual-frequency HIFU co-irradiation significantly increased the temperature of the focal region in the phantom in the lower power range (10 and 20 W) compared with single 1.1 MHz HIFU. It might be that dual-frequency HIFU irradiation can produce more cavitation bubbles and increase the energy absorption of the phantom [34,38]. At the same time, a large number of inertial cavitation can also increase heat production [34], significantly increasing the temperature at the low power of dual-frequency HIFU. Therefore, dual-frequency HIFU and activatable PFP@Polymer NPs showed their superiority in increasing thermal effects at lower power.

### 3.3. Evaluation of Anti-Tumor Efficiency of Dual-Frequency HIFU and Activatable PFP@Polymer NPs

**Figure 4:**
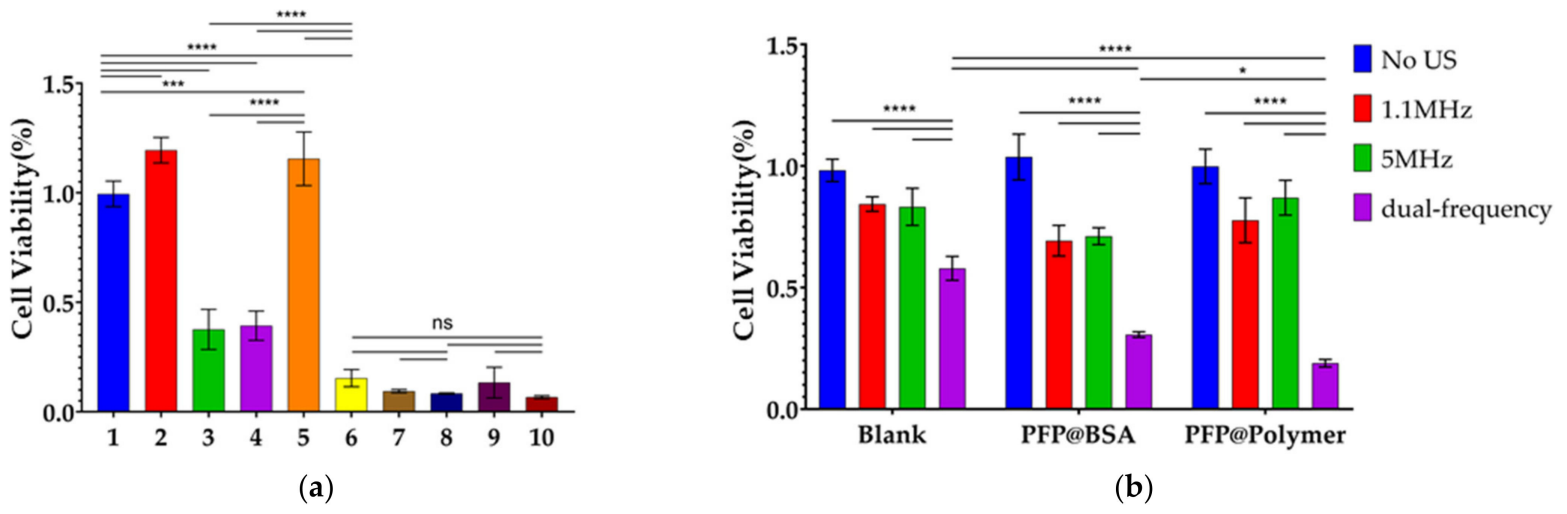
In vitro cell viability (**a**) after single-frequency and dual-frequency (both at 10 W) HIFU irradiation, with 1.1 MHz at various power values (10 W, 20 W, 30 W), by using cell-counting kit-8 (CCK-8) assay. The irradiation time of dual-frequency HIFU was 20 s; the power of 5 MHz HIFU was 10 W, and PFP@Polymer NPs were at the same volume concentration (0.002% *v*/*v*). 4T1 cells were divided randomly into 10 groups: (1) control, (2) only NPs (0.002% *v*/*v*), (3) 1.1 + 5 MHz US (10 + 10 W), (4) NPs + 1.1 MHz US (10 W), (5) NPs + 5 MHz US (10 W), (6) NPs + 1.1 MHz US + 5 MHz US (10 + 10 W), (7) 1.1 + 5 MHz US (20 + 10 W), (8) NPs + 1.1 MHz US + 5 MHz US (20 + 10 W), (9) 1.1 + 5 MHz US (30 + 10 W), (10) NPs + 1.1 MHz US + 5 MHz US (30 + 10 W). (**b**) Comparison of cell viability post 20 s irradiation of 1.1 MHz, 5 MHz, and dual-frequency HIFU in the following groups: blank (no addition of NDs or NPs), PFP@BSA (with the addition of the same volume concentration (0.002%) of PFP@BSA NDs), and PFP@Polymer (with the addition of the same volume concentration (0.002%) of PFP@Polymer NPs). * *p* < 0.05, *** *p* < 0.001, and **** *p* < 0.0001.

To evaluate the anti-tumor effect of PFP@Polymer NPs activated by dual-frequency HIFU irradiation in vitro, 4T1 cells were incubated with PFP@Polymer NPs and irradiated with single-frequency HIFU (1.1 or 5 MHz) and dual-frequency HIFU (1.1 and 5 MHz), respectively. Then cell viability was evaluated using a CCK-8 assay. The quantitative evaluation of the cell viability after different treatments is shown in Figure 4a. There was almost no cytotoxicity of PFP@Polymer NPs at the volume concentration of 0.002% (*v*/*w*) (Group 2), and there was almost no decrease in cell viability when irradiated by 5 MHz (10 W) HIFU alone (Group 5). Dual-frequency HIFU (both at 10 W) irradiation without NPs (Group 3) and single 1.1 MHz HIFU (10 W) irradiation with NPs (Group 4) produced similar therapeutic effects. Dual-frequency HIFU irradiation with NPs (Group 6) significantly reduced tumor cell activity with a high inhibition rate of 84.5% ± 3.4%. After increasing the power of 1.1 MHz in dual-frequency HIFU to 20 W and 30 W, dual-frequency HIFU itself could well inhibit the activity of tumor cells (Groups 7 and 9). Thus, there was no significant decrease in cell viability after adding NPs (Groups 8 and 10). There was also no significant difference between dual-frequency HIFU 20 W or 30 W and 10 W dual-frequency HIFU with NPs. This indicates that the treatment of dual-frequency HIFU at 10 W power combined with PFP@Polymer NPs can significantly reduce cell viability and ensure higher safety at lower power.

In addition, we compared the in vitro single-frequency and dual-frequency therapeutic effects of common nanodroplets PFP@BSA and our prepared PFP@Polymer NPs. A quantitative analysis of tumor cell activity is shown in Figure 4b. It shows that the curative effect of dual-frequency HIFU (both at 10 W) is limited. When adding PFP@BSA or PFP@Polymer, the therapeutic effect is significantly enhanced, and the cell viability is significantly decreased (**** *p* < 0.0001). However, single-frequency therapy had no obvious effect. Moreover, the therapeutic effects of PFP@BSA and PFP@Polymer showed the same trend, but the therapeutic effect of dual-frequency HIFU combined with the PFP@Polymer group was significantly better (* *p* < 0.05).

### 3.4. Evaluation of Enhanced Ultrasound Imaging In Vitro

**Figure 5:**
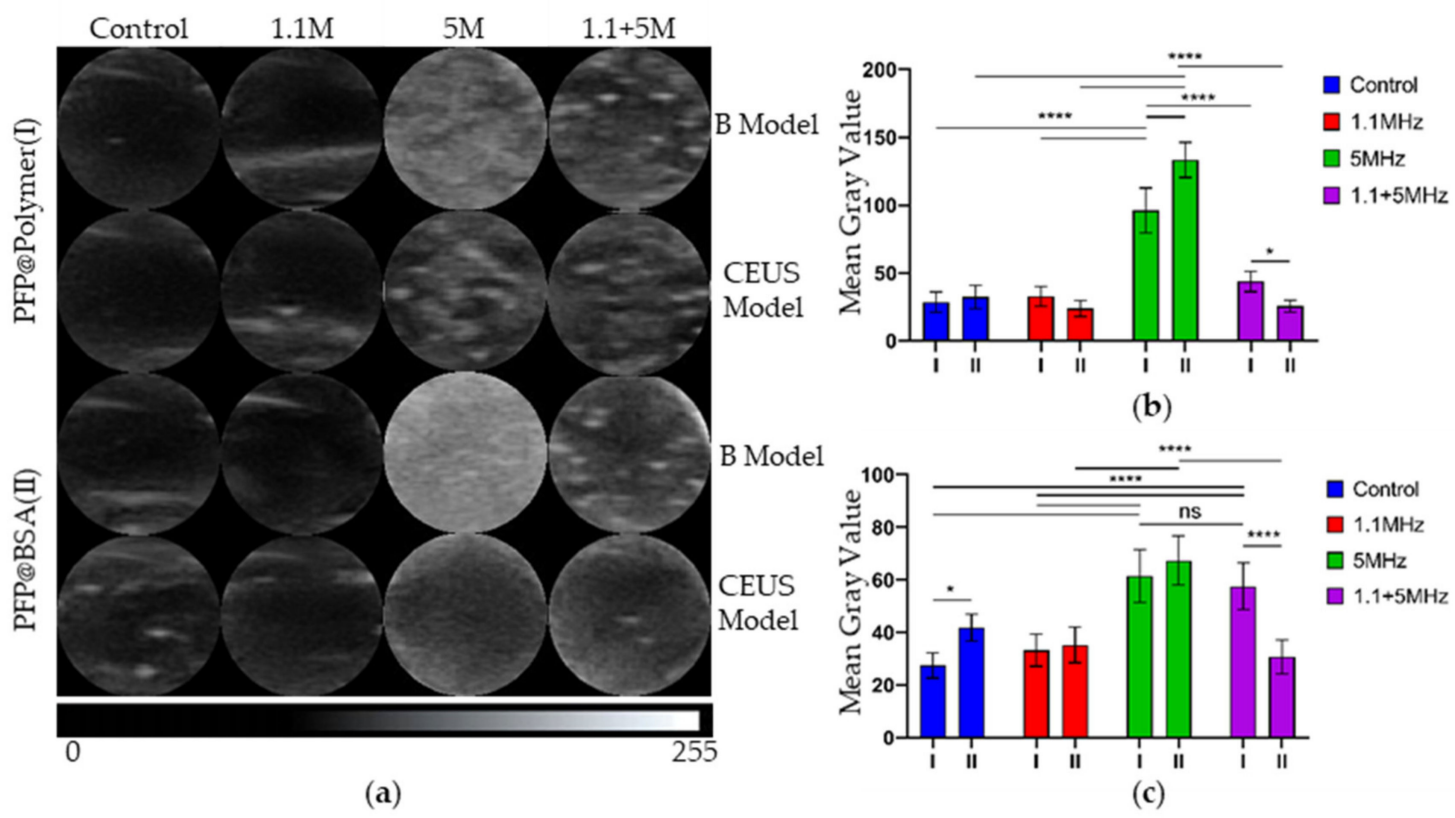
In vitro US imaging performance. (**a**) B-mode and contrast-enhanced ultrasound (CEUS) imaging of PFP@Polymer nanoparticles after 1.1 MHz, 5 MHz, and 1.1 + 5 MHz HIFU irradiation (1.1 MHz: 10 W, 6 ms, and 100 Hz; 5 MHz: 10 W, 100 μs, and 100 Hz) for 20 s in gel phantoms. The same concentration of PFP@BSA nanodroplets was used as that of controls. The mean US intensity values in both (**b**) B mode and (**c**) CEUS mode images with PFP@Polymer nanoparticles (I) and PFP@BSA nanodroplets (II) in different modes of HIFU irradiation. The diameter of the circular area is 1 cm. * *p* < 0.05 and **** *p* < 0.0001.

The US imaging capability of the microbubbles resulting from the single-frequency and dual-frequency HIFU activation of PFP@Polymer NPs was evaluated in vitro to confirm the potential of PFP@Polymer NPs as a US theranostic agent for US-imaging-guided therapy of tumors. Figure 5a shows the B mode and CEUS mode imaging after single-frequency and dual-frequency HIFU irradiation on PFP@Polymer NPs and PFP@BSA nanodroplets for 20 s, respectively. The mean US signal intensities in B mode and CEUS mode were calculated by Image J software, as presented in Figure 5b,c, showing that the gray value increases significantly after 5 MHz HIFU irradiation (**** *p* < 0.0001) but has no obvious change after 1.1 MHz HIFU irradiation. Compared with 1.1 MHz HIFU, 5 MHz HIFU can obviously enhance the cavitation effect, and a large number of cavitation microbubbles act as a contrast agent to enhance the gray value of the US image, which is consistent with the expectation. After dual-frequency HIFU irradiation, the gray value is lower than after 5 MHz HIFU irradiation alone. During dual-frequency irradiation, the cavitation and thermal effects are further enhanced, and a large number of microbubbles cause inertial cavitation to collapse or merge with each other to form larger bubbles floating out of the liquid surface, which might reduce the gray value of US imaging.

In both B mode and CEUS mode imaging, the gray value of PFP@Polymer NPs activated by dual-frequency HIFU irradiation was significantly stronger than that of PFP@BSA NDs, which was probably because the particle size of PFP@Polymer NPs is more uniform and stable than that of PFP@BSA NDs, which could produce more stable microbubbles. Moreover, CEUS mode is obviously more suitable to monitor the activated microbubbles here (**** *p* < 0.0001). In the CEUS mode imaging of our designed NPs, both dual-frequency HIFU and 5 MHz HIFU can significantly enhance the gray value of the image, which has a significant advantage over ordinary PFP@BSA NDs and shows great potential in the HIFU theranostics.

## 4. Discussion

Improving the efficiency of HIFU under lower and safer power is an urgent requirement in HIFU therapy. The PFP@Polymer NPs designed and prepared in this paper have the characteristics of stable structure and good biocompatibility and are HIFU activatable. Dual-frequency HIFU can effectively activate the phase transition of PFP into microbubbles, significantly reducing the thermal damage threshold of HIFU, enhancing its anti-tumor efficacy, and realizing ultrasound monitoring imaging. The potential mechanism of dual-frequency-HIFU-enhanced cavitation could be related with bubble dynamics [23]. A dual-frequency approach displays more resonances termed as “combination resonances” and could promote the acoustical scattering cross-section significantly within a much wider range of bubble sizes due to the generation of more resonances [32]. In this study, dual-frequency HIFU irradiation on phantoms and cells containing PFP@Polymer NPs produces microbubbles, which enhances the thermal and cavitation effect of HIFU, reduces the damage threshold, increases the damage volume, shortens the treatment time, and can also produce a significant temperature increase at lower power.

Cavitation is the formation and activity of a gas-filled bubble under acoustic excitation in a medium. The gas bubble could either oscillate stably or expand gradually and eventually collapse (stable and inertial cavitation). Cavitation could lead to thermal effects as well as chemical and optical effects [23]. Cavitation-enhanced anti-tumor efficacy mainly results from the enhanced thermal effects and mechanical/chemical effects of cavitation. Tung et al. [39] studied microbubble-enhanced heating in polyacrylamide phantoms embedded with different concentrations of Definity microbubbles (Lantheus Medical Imaging, North Billerica, MA, USA). They observed that microbubbles enhanced the heating in phantoms and reduced the power required to form a lesion by about 30% and hypothesized that microbubbles act as nucleation sites that reduce the threshold of inertial cavitation and thus contribute to heating enhancement. Clark et al. [20] also observed that the temperature in the tissue would suddenly increase significantly when cavitation bubbles appeared, and they proved that the inertial cavitation of microbubbles was the main reason for the rapid temperature rise. As an ultrasound contrast agent, microbubbles can increase the scattering and reflection of ultrasound in tissue, and they can also increase the absorption loss of ultrasound in tissue so as to increase heat production [40]. Moreover, inertial cavitation can overcome the limitation of short penetration distance of the EPR effect, improve the penetration ability of drugs, and enhance the permeability of cell membrane to increase the cell uptake of drugs [24]. Not only that, compared with the traditional PFP@BSA NDs, PFP@Polymer NPs have better anti-tumor efficacy (Figure 4) and better CEUS imaging effect (Figure 5), which might be related with the uniform and stable structure of PFP@Polymer NPs.

Contrast-enhanced ultrasound (CEUS) continues to gain traction as a technique that complements traditional B-mode and Doppler ultrasound in diagnostic and monitoring imaging [41]. A pulse inversion or amplitude modulation technique is used to reduce signals from normal tissues, which is akin to subtraction imaging in contrast-enhanced CT (CECT) or MRI. With the pulse inversion method, two sequential opposed-phase ultrasound pulses are transmitted into tissue, cancel out the linear echoes from background tissue, and leave only the enhanced nonlinear signal from the microbubbles. PFP phase transition to form microbubbles to enhance contrast-enhanced imaging is a common method of ultrasound monitoring imaging [29,30,42]. The imaging effect of CEUS is related to the concentration of microbubbles and the nonlinear vibration of microbubbles [43]. The high concentration of microbubbles produces a stronger gray scale, but the microbubble vibration, especially the collapse of microbubbles caused by the inertial cavitation vibration, will reduce the concentration of microbubbles and the imaging contrast. However, the inertial cavitation of microbubbles exerts a strong mechanical force on the tissue–cell interface [44], and it can also significantly increase the amount of ROS [22], thus enhancing the synergistic effect of cavitation in HIFU therapy. Therefore, there is a need for the balance of cavitation between cavitation imaging and the enhancement of HIFU. Under the action of HIFU with lower power in this paper, dual-frequency-ultrasound-enhanced microbubble cavitation met therapeutic efficiency and produced better CEUS imaging, indicating that the PFP@Polymer NPs have a great potential in further application in HIFU therapy at lower power.

Moreover, the internal use of PFP nanodroplets might be limited if they are unstable in body temperature. The boiling temperature of PFP is 29 °C. However, the vaporization boiling point of PFP droplets increases in relation to the droplet size and the shell coating material. Droplets with smaller diameters have higher Laplace pressure and are less easily vaporized into microbubbles [27,45]. For naked droplets 1 μm in size, the boiling point increases to 63 °C [46]. Thus, nanoscale droplets normally remain in a superheated state but will not vaporize into microbubbles until a minimum threshold acoustic pressure has been achieved or the temperature is higher than their boiling point [47]. However, nanodroplets in the bloodstream will be exposed in the strong pressure gradient while they pass through heart valves. PFP@Polymer nanoparticles might be vaporized by the large pressure modification and then cause shock to especially small animal models, even though we have used fluorinated PFPMA to enhance the stability of PFP droplets. Moreover, in our previous study on ADV, a small number of random nanodroplets were still vaporized at body temperature at the acoustic intensity lower than ADV threshold, which was probably because the small droplets aggregated into a large one, which decreased the boiling point and may affect the safety of our nanoparticles in vivo. In future research, replacing PFP with perfluorohexane (PFH) might be considered for the enhanced stability of nanoparticles.

Furthermore, the PFP@Polymer NPs prepared in this study contained several stable chemical bonds on the surface. So, they could work as a fundamental nanosystem to load more theranostic agents, such as SDT agents, chemotherapeutic agents, radiopharmaceutical, immunotherapeutic agents, MRI contrast agents, fluorescent particles, and photoacoustic imaging agents and realize the multifunctional and precise theranostics aimed at special targets in several diseases, including tumors, brain diseases, and functional disorders. PFP@Polymer NPs show a great potential in further preclinical and clinical applications.

## 5. Conclusions

An activatable PFP-loaded polymer nanoparticle was successfully constructed in this study. Under the irradiation of dual-frequency (1.1 and 5 MHz) HIFU, the phase transition of PFPs into microbubbles was realized, which play an excellent role as ultrasound contrast agents and cavitation nuclei for enhanced HIFU theranostics. Combined with PFP@Polymer NPs, dual-frequency HIFU reduced the thermal damage threshold significantly, produced higher temperature increase, and exhibited a higher tumor inhibition rate at low power in vitro than the single-frequency HIFU. Moreover, benefitting from their uniform and stable structure, PFP@Polymer NPs showed higher anti-tumor efficacy and better CEUS imaging ability than the traditional PFP@BSA NDs. PFP@Polymer NPs could also be modified with theranostic agents on their surface, laying a foundation for further applications of multifunctional HIFU theranostics.

## Figures and Tables

**Table 1:** Acoustic intensity of 1.1 MHz high-intensity focused ultrasound (HIFU) at different electric powers.

*P* * (W)	10	20	30	40	50	60	70	80	90	100	110	120	130	140
*I*_1.1 MHz_ # $(W/{\mathrm{cm}}^{2}$)	23.8	43.1	62.4	81.7	101.0	120.3	139.6	158.9	178.2	197.6	216.9	236.2	255.5	275.8

* *P* is the electric power (W), # *I*_1.1 MHz_ is the acoustic intensity of 1.1 MHz HIFU (W/cm^2^).

**Table 2:** Acoustic intensity of 5 MHz HIFU at different electric powers.

*P* * (W)	10	20	30	40	50	60
*I*_5 MHz_ # $(W/{\mathrm{cm}}^{2}$)	0.35	0.85	1.55	2.48	3.61	4.96

* *P* is the electric power (W), # *I*_5 MHz_ is the acoustic intensity of 5 MHz HIFU (W/cm^2^).

**Table 3:** Acoustic threshold for 1.1 MHz HIFU with and without PFP@Polymer NPs.

	Power of Single Frequency 1.1 MHz HIFU (W)								
	20	30	40	60	80	100	110	120	130
Without NPs (40 s)	×	×	×	×	×	×	×	√	√
With NPs (20 s)	×	√	√	√	√	√	√	√	√

(√) can produce damage lesions; (×) cannot produce damage lesions.

**Table 4:** Acoustic threshold for the single and dual frequency HIFU with PFP@Polymer NPs.

	Power Gradient (W)					
	10	20	30	40	50	60
Dual-Frequency (5 MHz:10W; 1.1 MHz)	√	√	√	√	√	√
1.1 MHz	×	×	√	√	√	√
5 MHz	×	×	×	×	×	×

(√) can produce damage lesions; (×) cannot produce damage lesions.
